# Systematic Characterization and Longitudinal Study Reveal Distinguishing Features of Human Milk Oligosaccharides in China

**DOI:** 10.1093/cdn/nzaa113

**Published:** 2020-07-02

**Authors:** Jiayi Wu, Shaohui Wu, Jinhong Huo, Hongbo Ruan, Xiaofei Xu, Zhanxi Hao, Yuan'an Wei

**Affiliations:** Quantum Hi-Tech (China) Biological Co., Ltd, Jiangmen City, Guangdong Province, People's Republic of China; Quantum Hi-Tech (China) Biological Co., Ltd, Jiangmen City, Guangdong Province, People's Republic of China; Quantum Hi-Tech (China) Biological Co., Ltd, Jiangmen City, Guangdong Province, People's Republic of China; Quantum Hi-Tech (China) Biological Co., Ltd, Jiangmen City, Guangdong Province, People's Republic of China; Quantum Hi-Tech (China) Biological Co., Ltd, Jiangmen City, Guangdong Province, People's Republic of China; Quantum Hi-Tech (China) Biological Co., Ltd, Jiangmen City, Guangdong Province, People's Republic of China; Quantum Hi-Tech (China) Biological Co., Ltd, Jiangmen City, Guangdong Province, People's Republic of China

**Keywords:** human milk oligosaccharides, Chinese mothers, secretor, nonsecretor, longitudinal study, HPAEC, dynamic change, profile features

## Abstract

**Background:**

Human milk oligosaccharides (HMOs) in breast milk contribute to the development of the neonatal microbiota and immune system. However, longitudinal studies examining HMO profiles of Chinese mothers remain scarce.

**Objectives:**

We aimed to analyze HMO profiles, including their composition, concentrations, and changes during lactation, in milk of Chinese mothers.

**Methods:**

A total of 822 milk samples from 222 mothers were collected, of which 163 mothers provided single samples. Samples from the remaining 59 mothers were collected on day 3, day 7, and thereafter every 7 or 14 d until day 168. 24 HMOs were studied using high-performance anion-exchange chromatography. Secretor and nonsecretor status were determined based on Lewis blood types and a defined 2′-fucosyllactose (2′-FL) threshold.

**Results:**

Of the 222 mothers, 77% were secretors and 23% were nonsecretors. The longitudinal study involving 59 mothers showed that the total HMOs in secretors were significantly greater than those in nonsecretors during the first 2 wk. Acidic HMOs decreased significantly during lactation and were similar between secretors and nonsecretors. Among neutral HMOs, distinctive differences were observed. Nonfucosylated and α-1-3/4-fucosylated HMOs in nonsecretors were significantly higher than those in secretors during the first month. In contrast, α-1-2-fucosylated HMOs in secretors were significantly higher than those in nonsecretors throughout 168 d. In secretors, 2′-FL concentrations peaked at (mean ± SEM) 3.02 ± 0.14 g/L (day 3) followed by significant decreases. In nonsecretors, 2′-FL concentrations were fairly low throughout 168 d. Of the 24 studied HMOs, only 3-fucosyllactose concentrations increased during lactation in both secretor and nonsecretor mothers.

**Conclusions:**

Our study showed dynamic changes of 24 HMOs in secretors and nonsecretors during lactation and revealed unique features of these HMO profiles in the milk of Chinese mothers. Interestingly, 2′-FL concentrations in secretors were found to be lower than those of Western populations but higher than those of African populations.

## Teaser

A systematic characterization and longitudinal study of HMOs in secretor and nonsecretor Chinese mothers reveals overlapping and distinctive features compared with HMO profiles around the world.

## Introduction

Human milk oligosaccharides (HMOs) are the third most abundant solid component in human milk and are important natural prebiotics that contribute to healthy neonatal microbial colonization in the gastrointestinal tract (1–4). They have been shown to modulate intestinal epithelial cell function and immune responses by regulating gene expression and cytokine production (5–9). In addition, HMOs act as antibacterial agents that inhibit pathogenic microbial adhesions and reduce intestinal infections such as necrotizing enterocolitis (10–13). They also serve as a source of sialic acid, which is an essential nutrient for brain growth and cognitive development in newborns (14, 15).

HMO composition varies greatly among mothers owing to factors such as genetic polymorphisms found in the fucosyltransferase 2 (*FUT2*, *Se* gene) and fucosyltransferase 3 genes (*FUT3*, *Lewis* (*Le*) gene) (16). The *FUT2* gene encodes fucosyltransferase (FucT) II and mothers can be classified as secretors (*Se+*) or nonsecretors (*Se–*) based on its level of expression (17). FucT II transfers fucose to terminal galactose in an (α-1-2)-linkage (14). Its expression contributes to the diverse products of α-1-2-fucosylated HMOs in the milk of secretor mothers, whereas its absence in nonsecretor mothers results in little to no production of α-1-2-fucosylated HMOs (14, 18–21). Therefore, concentrations of the α-1-2-fucosylated HMO 2′-fucosyllactose (2′-FL) have commonly been used to define secretor and nonsecretor types of mothers (20–22). The *FUT3* (*Le*) gene encodes FucT III, which catalyzes the transfer of fucose by an (α-1-4)-linkage (17). Expression of the *FUT2* (*Se*) and *FUT3* (*Le*) genes results in the diverse Lewis blood types. Among *Le+* blood types, Le(a+b−) individuals are known as *Se*− nonsecretors whereas Le(a−b+) individuals are *Se+* secretors. Individuals of Le(a+b+) blood type are *Se^weak^Le+* partial secretors (23). For *Le*− individuals, Le(a−b−) can be either *Se+* secretors or *Se*− nonsecretors (24, 25). As a result, Le(a+b−) and Le(a−b+) Lewis blood types can be used to directly identify nonsecretor and secretor types of mothers, respectively.

In addition to maternal genotype of FucTs, geography and lactation stage are also important contributors to variation in HMO profiles (16, 20, 26). Different percentages of secretor mothers have been reported in different regions around the world (20). In the United States, the percentage of secretor-type mothers was 68% in Washington State (20). In Europe, it was reported to be 76% in Spain and 79% in Sweden (20). In Africa, it was 68% in Ghana and 81% in Kenya (20). Another factor that has been shown to influence changes of HMOs is the lactation stage (26, 27). Commonly, breast milk during days 0–5 of lactation is considered colostrum, from day 6 to day 30 it is considered transitional milk, and after 30 d, it is regarded as mature milk (28). Most colostrum contains the highest amounts of total HMOs, whereas in transitional and mature milk, concentrations of 2′-FL decreased but those of 3-fucosyllactose (3-FL) increased (29).

However, to date, most research on HMO profiles has been performed in European and American countries (27). There are limited data from Asian, especially Chinese, populations regarding HMO profiles and their dynamic changes in mothers’ milk throughout lactation. Recent advancements in high-performance anion-exchange chromatography with pulsed amperometric detection (HPAEC-PAD) have allowed for a more accurate and sensitive detection of different oligosaccharides in hydroxyl dissociation under alkaline conditions without derivatization (30, 31). In the present study, HPAEC-PAD was used to simultaneously quantify 24 HMOs in human milk, including 17 neutral and 7 acidic HMOs. Specifically, 822 milk samples from 222 Chinese mothers were analyzed, and secretor status was defined based on 2′-FL concentrations. Of these mothers, 59 were continually sampled at day 3, day 7, and thereafter at intervals of 7 or 14 d in order to investigate changes of HMO profiles throughout the lactation period. The results from Chinese mothers were compared with earlier HMO publications worldwide to examine the variation in HMO profiles among people of different races and geographical regions.

## Methods

### Study design

This study was conducted from March 2015 to July 2019 with volunteers recruited mainly from Jiangmen and Guangzhou, cities of Guangdong Province, PR China. It is the initial effort to build an HMO database of Chinese mothers. All experimental design, implementation, and analyses were performed according to related scientific guidelines. Volunteers were healthy 23- to 39-y-old mothers, all of whom signed voluntary consent forms. Study design and procedures were conducted according to working protocols, which were reviewed and advised by specialists from local hospitals and approved by the ethical committee of Quantum Hi-Tech (China) Biological Co., Ltd. Questionnaires were used to gather basic information about mothers and infants, including maternal age, height, and weight, weeks of pregnancy, mode of delivery, parity, and the newborn's birth date, height, weight, and gender. Exclusionary criteria included gestational diabetes, hypertension, cardiovascular diseases, acute infectious diseases, and/or blood transfusions within 6 mo of recruitment.

**FIGURE 1:**
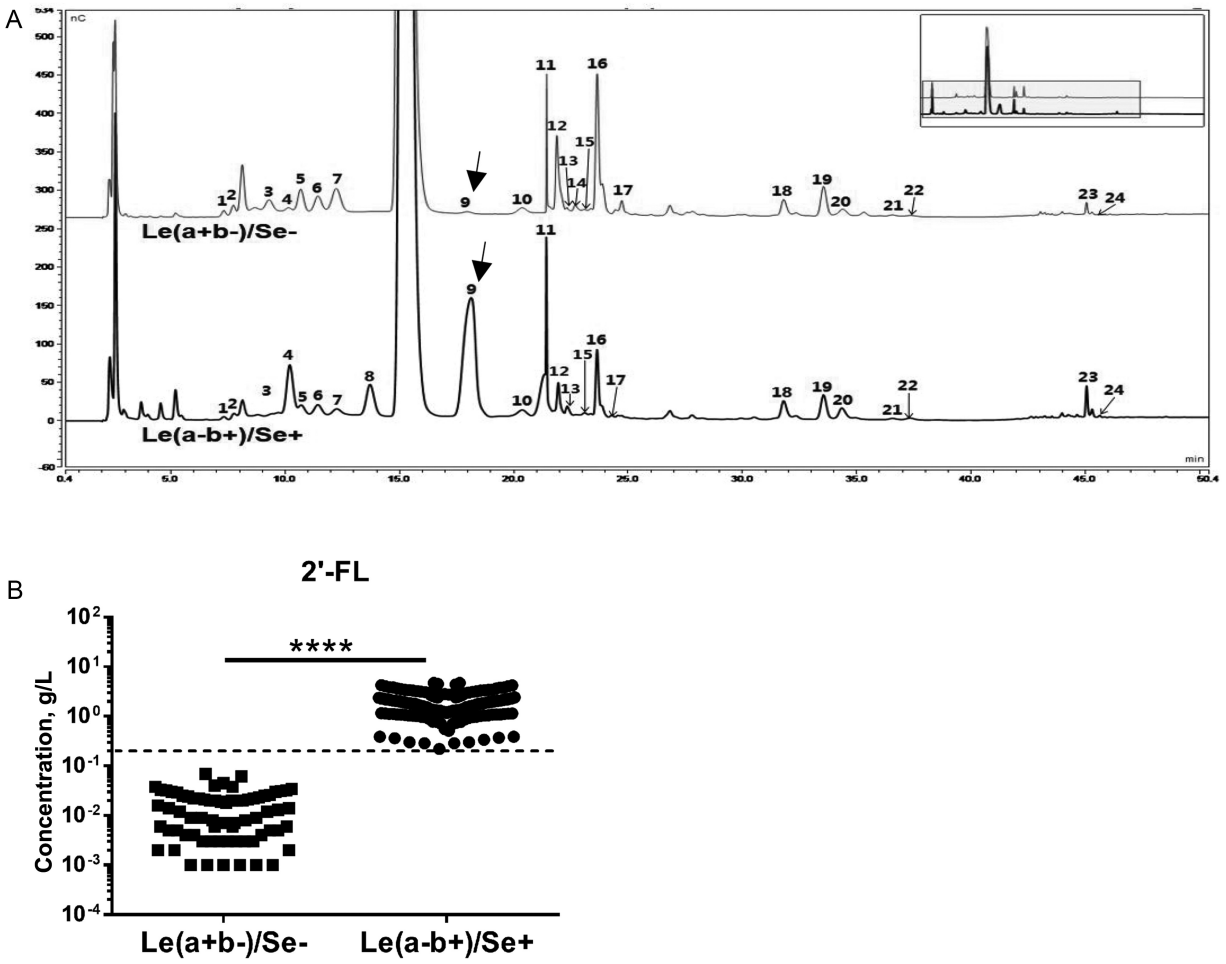
HMOs in milk samples of Le(a+b−)/Se− and Le(a−b+)/Se+ mothers. (A) Representative high-performance anion-exchange chromatography chromatograms of 24 HMOs in milk samples of Le(a+b−)/Se− and Le(a−b+)/Se*+* mothers. Arrows indicate peak 9 of 2′-FL. 1. Lacto-N-neodifucohexaose, 2. LNDFH II, 3. Difucosyllacto-N-hexaose, 4. LNDFH I, 5. 3-fucosyllactose, 6. Blood Group A tetrasaccharide, 7. LNFP II, 8. Lactodifucotetraose, 9. 2′-FL, 10. Lacto-N-triose, 11. LNFP I, 12. Lacto-N-neotetraose, 13. Lacto-N-neofucopentaose, 14. LNFP V, 15. p-Lacto-N-neohexaose + lacto-N-neohexaose, 16. Lacto-N-tetraose, 17. Lacto-N-neooctaose, 18. LSTc, 19. 6′-Sialyllactose, 20. 3′-Sialyllactose, 21. LSTa, 22. LSTb, 23. Disialyllacto-N-tetraose, 24. Disialyl lactose. (B) 2′-FL concentrations in milk of Le(a+b−)/Se− and Le(a−b+)/Se*+* mothers. Dashed lines indicate 0.2 g/L. ^****^P < 0.0001. HMO, human milk oligosaccharide; LNDFH, lacto-N-difucohexaose; LNFP, lacto-N-fucopentaose; LST, LS-tetrasaccharide; Se+, secretor; Se−, nonsecretor; 2′-FL, 2′-fucosyllactose.

The study consisted of 2 parts. In the first part, single samples were collected from 163 mothers within 168 d of lactation to determine the percentage of secretor mothers. In the second part, 59 mothers were similarly recruited and continually sampled until 168 d of lactation to analyze the dynamic changes of their HMO profiles. Specifically, colostrum was collected on day 3 of lactation. Thereafter, milk samples were collected every 7 or 14 d from day 7 of lactation until the cessation of breastfeeding, with the latest sampling time at day 168. **Supplemental Table 1** summarizes characteristics of these 59 mothers and their infants. Concentrations of 24 HMOs were determined in each sample and **Table 1** summarizes the structures of these 24 HMOs. Based on 2′-FL concentrations in the Le(a+b−) and Le(a−b+) blood types (known as nonsecretors and secretors, respectively), a threshold concentration of 0.2 g/L was chosen to define the secretor and nonsecretor types (**Figure 1**B). Mothers with a 2′-FL concentration >0.2 g/L in their milk were identified as secretors.

**TABLE 1:** Glycan structures of tested HMOs^1^

HMO	Glycan	Structure
LNnDFH	Lacto-N-neodifucohexaose	Galβ1-4(Fucα1-3)GlcNAcβ1-3Galβ1-4(Fucα1-3)Glc
LNDFH II	Lacto-N-difucohexaose II	Galβ1-3(Fucα1-4)GlcNAcβ1-3Galβ1-4(Fucα1-3)Glc
DFLNH	Difucosyllacto-N-hexaose	Galβ1-4(Fucα1-3)GlcNAcβ1-6[Galβ1-3(Fucα1-4)GlcNAcβ1-3]Galβ1-4Glc
LNDFH I	Lacto-N-difucohexaose I	Fucα1-2Galβ1-3(Fucα1-4)GlcNAcβ1-3Galβ1-4Glc
3-FL	3-Fucosyllactose	Galβ1-4(Fucα1-3)Glc
A-Type-6	Blood Group A tetrasaccharide	GlcNAcα1-3(Fucα1-2)Galβ1-4Glc
LNFP II	Lacto-N-fucopentaose II	Fucα1-4(Galβ1-3)GlcNAcβ1-3Galβ1-4Glc
LDFT	Lactodifucotetraose	Fucα1-2Galβ1-4(Fucα1-3)Glc
2′-FL	2′-Fucosyllactose	Fucα1-2Galβ1-4Glc
LNT2	Lacto-N-triose	GlcNAcβ1-3Galβ1-4Glc
LNFP I	Lacto-N-fucopentaose I	Fucα1-2Galβ1-3GlcNAcβ1-3Galβ1-4Glc
LNnT	Lacto-N-neotetraose	Galβ1-4GlcNAcβ1-3Galβ1-4Glc
LNnFP	Lacto-N-neofucopentaose	Galβ1-4GlcNAcβ1-3Galβ1-4(Fucα1-3)Glc
LNFP V	Lacto-N-fucopentaose V	Galβ1-3GlcNAcβ1-3Galβ1-4(Fucα1-3)Glc
p-LNnH*	p-Lacto-N-neohexaose	Galβ1-4GlcNAcβ1-3Galβ1-4GlcNAcβ1-3Galβ1-4Glc
LNnH*	Lacto-N-neohexaose	Galβ1-4GlcNAcβ1-6(Galβ1-4GlcNAcβ1-3)Galβ1-4Glc
LNT	Lacto-N-tetraose	Galβ1-3GlcNAcβ1-3Galβ1-4Glc
LNnO	Lacto-N-neooctaose	Galβ1-4GlcNAcβ1-3Galβ1-4GlcNAcβ1-3Galβ1-4GlcNAcβ1-3Galβ1-4Glc
LSTc	LS-tetrasaccharide c	NeuAcα2-6Galβ1-4GlcNAcβ1-3Galβ1-4Glc
6′-SL	6′-Sialyllactose	NeuAcα2-6Galβ1-4Glc
3′-SL	3′-Sialyllactose	NeuAcα2-3Galβ1-4Glc
LSTa	LS-tetrasaccharide a	NeuAcα2-3Galβ1-3GlcNAcβ1-3Galβ1-4Glc
LSTb	LS-tetrasaccharide b	NeuAcα2-6(Galβ1-3)GlcNAcβ1-3Galβ1-4Glc
DSLNT	Disialyllacto-N-tetraose	NeuAcα2-6(NeuAcα2-3Galβ1-3)GlcNAcβ1-3Galβ1-4Glc
DS-L	Disialyl lactose	NeuAcα2-8NeuAcα2-3Galβ1-4Glc

^1^Fuc, fucose; Gal, galactose; Glc, glucose; GlcNAc, N-acetyl-glucose; HMO, human milk oligosaccharide; LNnH, lacto-N-neohexaose; NeuAc, N-acetyl-neuraminic acid; p-LNnH, p-Lacto-N-neohexaose.

* p-LNnH and LNnH were not separated well in our high-performance anion-exchange chromatography system. Thus, their concentrations are given as the sum of p-LNnH + LNnH.

### Collection and storage of milk samples

Breast milk was collected between 09:00 and 13:00 with ≥2 h of no feeding or breast pumping before collection. Unilateral breast milk was collected by milk pump or manual extrusion. After mixing, 5 mL of milk samples was stored in a centrifuge tube, properly labeled with the collection date and donor information, and then immediately frozen at −20°C. The frozen samples were transported in cold storage bags surrounded by ice packs to the laboratory and stored at −80°C. HMO analyses were performed within 6 mo of collection.

### Pretreatment of milk samples

In preparing for analysis, milk samples were thawed at room temperature. One milliliter of milk was mixed with 1 mL absolute ethanol and stored at 4°C to precipitate proteins and lipids for 60 min before centrifugation at 4°C for 20 min at 6,800 × *g*. After discarding the supernatant, ethanol was removed by nitrogen blow down. The remaining material was diluted with distilled water to 50 mL and then filtered through a 0.22-μm membrane before being stored at −20°C until HMO analysis within 1 wk.

### Analysis of HMOs in milk samples

HMOs in milk samples were quantified using HPAEC-PAD with an ICS-5000+ ion chromatography detection system (Thermo Scientific). This system was comprised of a CarboPac PA-1 (4 × 250 mm, 6.5 μm) column, a CarboPac PA-1 (4 × 50 mm) precolumn, and a pulsed amperometric detector with a gold electrode. The analysis was performed according to a previously published method with minor modifications (29). HMO standards were included as controls to quantitatively calculate concentrations of 24 HMOs in the milk samples. All HMO standards were purchased from Carbosynth. Table 1 summarizes details of the 24 HMOs tested.

### Collection and storage of blood samples

Fifty-microliter blood samples were collected from the fingertips of volunteers using disposable sterile needles. Two milliliters of self-prepared Alsever's solution (32) was added immediately, then the samples were transported to the laboratory in frozen storage bags containing ice packs. Samples were stored at 4°C and analyzed for Lewis blood type within 24 h.

### Pretreatment of blood samples

Blood samples were centrifuged at 4°C for 1 min at a speed of 100 × *g*. After supernatants were discarded, blood cells were resuspended in 1 mL normal saline (0.9% NaCl solution), then centrifuged again using the aforementioned conditions. Blood cells were resuspended to 0.5% (vol:vol) with normal saline for testing.

### Lewis blood type identification

Lewis blood group was identified using an agglutination test kit of RBCs (CE-Immundiagnostika GmbH). Procedures were performed according to the manufacturer's guidelines. Briefly, anti-Le^a^ and anti-Le^b^ sera reagents were each mixed with equal volumes of the aforementioned prepared 0.5% blood cell suspension and allowed to stand at room temperature for 15 min. After centrifugation for 1 min at 100 ×* g*at room temperature, the blood cells were resuspended with normal saline. Finally, 50 μL of the resuspension was placed in a 96-well plate and observed under a microscope. If agglutination occurred in the antiserum mixture, the corresponding Le antigen was recorded as positive in the sample. The absence of agglutination was recorded as negative of that antigen.

### Statistical analysis

Data analyses were performed using SPSS statistics software version 17.0 (IBM Corp.) and GraphPad Prism version 6 (GraphPad Software, Inc.). To determine significant differences of HMOs in milk from secretor and nonsecretor mothers, independent-samples *t* test, unpaired *t* test, and multiple *t* test were used accordingly. To determine significant differences at different time points during the lactation period, a 1-factor ANOVA followed by the Student–Newman–Keuls test was used. Values were expressed as means ± SEMs unless otherwise stated and a *P* value < 0.05 was considered significant.

## Results

### HMO differences among Chinese mothers of various Lewis blood types

Among the 59 continually followed mothers, the Lewis blood types of 24 mothers were identified directly by serological tests. Of the 24 mothers, 14 (58%) had a Lewis blood type of Le(a−b+), 5 (21%) Le(a+b−), 4 (17%) Le(a−b−), and 1 (4%) Le(a+b+). Figure 1A shows representative HPAEC chromatograms of the 24 tested HMOs in day 3 colostrum from Le(a−b+)/*Se+* and Le(a+b−)*/Se*− mothers. Le(a−b+) secretor mothers had breast milk that was rich in α-1-2-fucosylated HMOs such as lacto-N-difucohexaose (LNDFH) I, lactodifucotetraose (LDFT), 2′-FL, and lacto-N-fucopentaose (LNFP) I (peaks 4, 8, 9, and 11) (Figure 1A). In contrast, the Le(a+b−) nonsecretor mothers had little to no α-1-2-fucosylated HMOs. However, higher concentrations of α-1-3/4-fucosylated HMOs such as 3-FL and LNFP II (peaks 5 and 7) were found in the Le(a+b−) nonsecretor mothers than in the Le(a−b+) secretor mothers (Figure 1A).

Figure 1B shows that 2′-FL concentrations were significantly higher in the breast milk of Le(a−b+) secretors than in that of Le(a+b−) nonsecretors. Interestingly, 2′-FL concentration at 0.2 g/L separated Le(a−b+) secretors and Le(a+b−) nonsecretors with 100% accuracy compared to serological tests (analyzed by receiver operating characteristic statistical analysis, data not shown). Based on the 0.2 g/L 2′-FL threshold, 14 Le(a−b+) mothers and 3 out of 4 Le(a−b−) mothers were classified as secretors, whereas the 5 Le(a+b−) mothers, 1 Le(a+b+) mother, and remaining 1 Le(a−b−) mother were classified as nonsecretors.

Representative HPAEC chromatograms of the 24 tested HMOs in day 3 colostrum from all 5 Lewis/secretor types were summarized (**Supplemental Figure 1**A). Interestingly, although qualified as a nonsecretor (with 2′-FL concentrations <0.2 g/L), 2′-FL (peak 9) in the Le(a+b+) mother was modestly produced (Supplemental Figure 1A). However, it is worth noting that such 2′-FL concentrations in the breast milk of the Le(a+b+) mother were significantly higher than those of other nonsecretors, but were significantly lower than those in secretors of Le(a−b+) phenotype (Supplemental Figure 1B). Therefore, the Le(a+b+) mother was classified as a partial secretor, which was also suggested by another group (23). Among nonsecretor mothers, lacto-N-tetraose (LNT) concentrations in Le(a−b−) mothers were significantly higher than those in Le(a+b−) and Le(a+b+) mothers (Supplemental Figure 1C).

### Distinct features of HMOs in secretor and nonsecretor types of Chinese mothers during lactation

All milk samples were grouped into secretor and nonsecretor types according to 2′-FL concentrations (secretors > 0.2 g/L and nonsecretors < 0.2 g/L) (Figure 1B). Mothers who donated samples of secretor milk were classified as secretor mothers. Among the 222 mothers, 171 (77%) were found to be secretors whereas 51 (23%) were nonsecretors. Significantly higher concentrations of 2′-FL were found in milk of secretor mothers than in that of nonsecretor mothers (**Supplemental Figure 2**). Similar significant differences between secretor and nonsecretor mothers were found for other α-1-2-fucosyltransferase products such as LDFT, LNDFH I, and LNFP I (Supplemental Figure 2).

**FIGURE 2:**
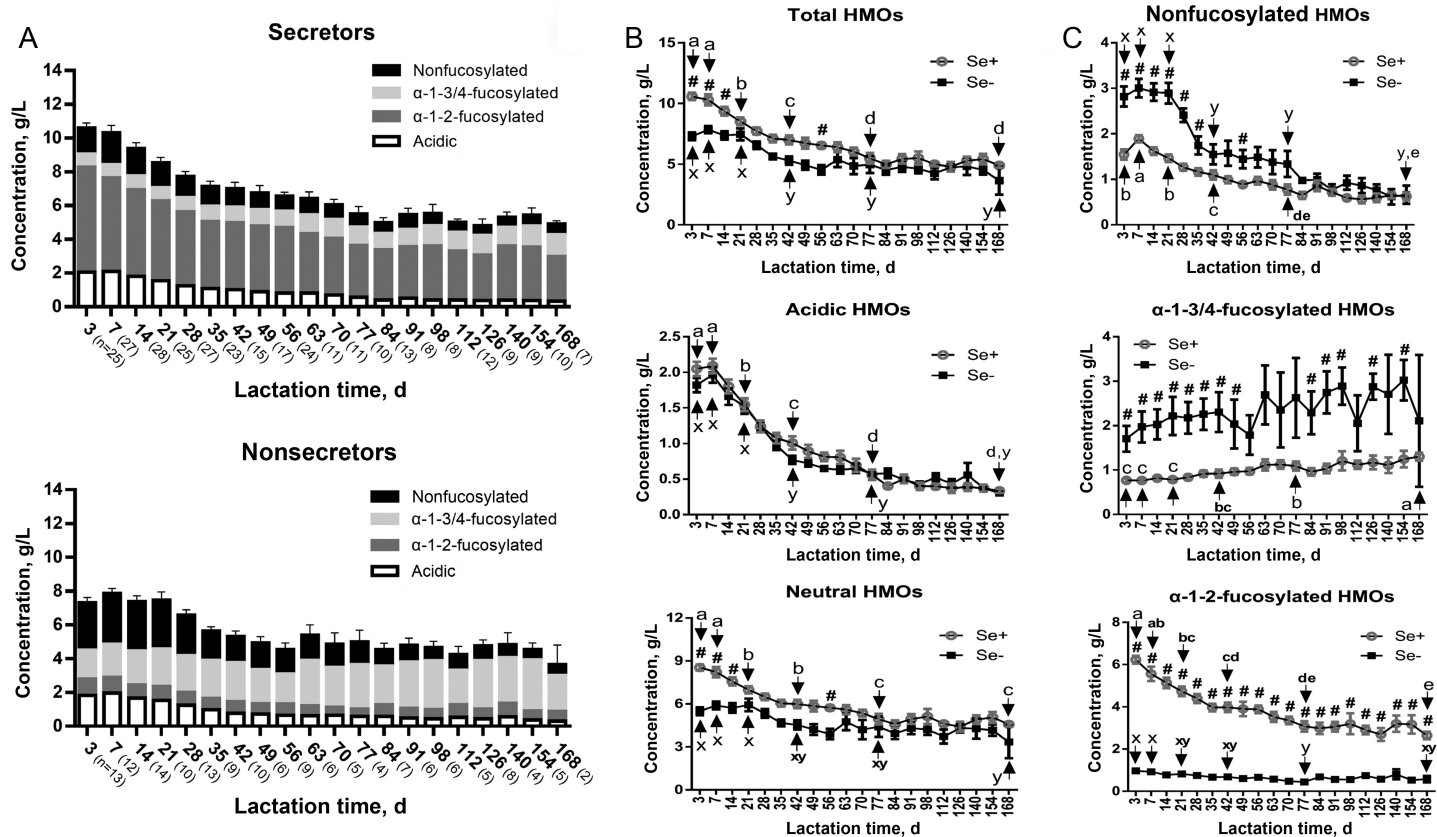
Concentration changes of HMOs in milk of Se+ and Se− mothers during lactation. (A) Overview of HMO subtype changes in Se+ and Se− mothers during lactation. Acidic HMOs are calculated as LSTc + 6′-sialyllactose + 3′-sialyllactose + LSTa + LSTb + disialyllacto-N-tetraose + disialyl lactose. Nonfucosylated HMOs are calculated as lacto-N-triose + lacto-N-neotetraose + p-lacto-N-neohexaose + lacto-N-neohexaose + lacto-N-tetraose + lacto-N-neooctaose. α-1-2-fucosylated HMOs are calculated as LNDFH I + Blood Group A tetrasaccharide + lactodifucotetraose + 2′-FL + LNFP I. α-1-3/4-fucosylated HMOs are calculated as lacto-N-neodifucohexaose + LNDFH II + difucosyllacto-N-hexaose + 3-fucosyllactose + LNFP II + lacto-N-neofucopentaose + LNFP V. Comparison of dynamic changes of (B) HMO concentrations and (C) neutral HMO subtypes in Se*+* and Se− mothers during lactation. All values are means ± SEMs. “a”, “b”, … and “x”, “y” … indicate differences among different time points in Se*+* and Se− mothers, respectively. # indicates statistical significance between Se*+* and Se− mothers at the same lactation time. *P* < 0.05 was considered significant. HMO, human milk oligosaccharide; LNDFH, lacto-N-difucohexaose; LNFP, lacto-N-fucopentaose; LST, LS-tetrasaccharide; Se+, secretor; Se−, nonsecretor.

To analyze changes of HMO profiles in secretors and nonsecretors during lactation, milk samples were continually collected from 59 healthy Chinese mothers from day 3 to day 168 of lactation. Distinctive features of HMO composition in milk of secretor and nonsecretor mothers were observed, especially for the 3 subtypes of neutral HMOs (nonfucosylated, α-1-3/4-fucosylated, and α-1-2-fucosylated) (**Figure 2**A). Total HMOs in milk of secretor mothers on day 3 (*n* = 25), day 7 (*n* = 27), and day 14 (*n* = 28) of lactation were all significantly higher than those in their nonsecretor counterparts (*n* = 13, 12, and 14, respectively) (Figure 2B). In milk of secretors and nonsecretors, concentrations of acidic HMOs were similar and differences in neutral HMO concentrations contributed to the observed differences in total HMOs (Figure 2B). Among the neutral HMO subtypes, both nonfucosylated and α-1-3/4-fucosylated HMOs were consistently higher in milk of nonsecretors than in that of secretors (Figure 2C). These differences were continuously significant until day 35 for nonfucosylated HMOs and until day 49 for α-1-3/4-fucosylated HMOs during lactation (Figure 2C). In contrast, α-1-2-fucosylated HMOs were significantly higher in milk of secretor mothers than in that of nonsecretors throughout the studied 168 d of lactation (Figure 2C).

### Dynamic changes of HMOs in secretor and nonsecretor types of Chinese mothers during lactation

To analyze dynamic changes of HMOs in milk of secretor and nonsecretor mothers, days 3, 7, 21, 42, 77, and 168 of lactation were selected to statistically test the HMO changes throughout lactation. As Figure 2B shows, the mean amount of total HMOs in secretors decreased significantly from 10.59 ± 0.30 g/L (day 3) to 4.90 ± 0.18 g/L (day 168), whereas in nonsecretors, it decreased significantly from 7.31 ± 0.32 g/L (day 3) to 3.65 ± 1.15 g/L (day 168). For acidic HMOs, similar significant decreases in milk of secretors and nonsecretors were observed during lactation (Figure 2B). Mean neutral HMOs significantly decreased from 8.54 ± 0.22 g/L (day 3) to 4.57 ± 0.16 g/L (day 168) in secretors, and from 5.49 ± 0.28 g/L (day 3) to 3.35 ± 1.13 g/L (day 168) in nonsecretors (Figure 2B). Among neutral HMOs, mean nonfucosylated HMOs in secretors initially increased significantly from 1.54 ± 0.11 g/L (day 3) to 1.89 ± 0.08 g/L (day 7) but thereafter significantly decreased to 0.62 ± 0.09 g/L (day 168) (Figure 2C). In milk of nonsecretors, mean nonfucosylated HMOs significantly decreased from 2.82 ± 0.22 g/L at day 3 to 0.66 ± 0.20 g/L at day 168 (Figure 2C). Increasing trends of mean α-1-3/4-fucosylated HMOs were found in milk of both secretors and nonsecretors, but statistical significance was only observed in secretors (from 0.77 ± 0.05 g/L at day 3 to 1.30 ± 0.09 g/L at day 168) (Figure 2C). α-1-2-fucosylated HMOs remained low with minor changes in milk of nonsecretors (Figure 2C). However, in milk of secretors, mean α-1-2-fucosylated HMOs started high but decreased significantly from 6.23 ± 0.19 g/L (day 3) to 2.64 ± 0.18 g/L (day 168) (Figure 2C).

**FIGURE 3:**
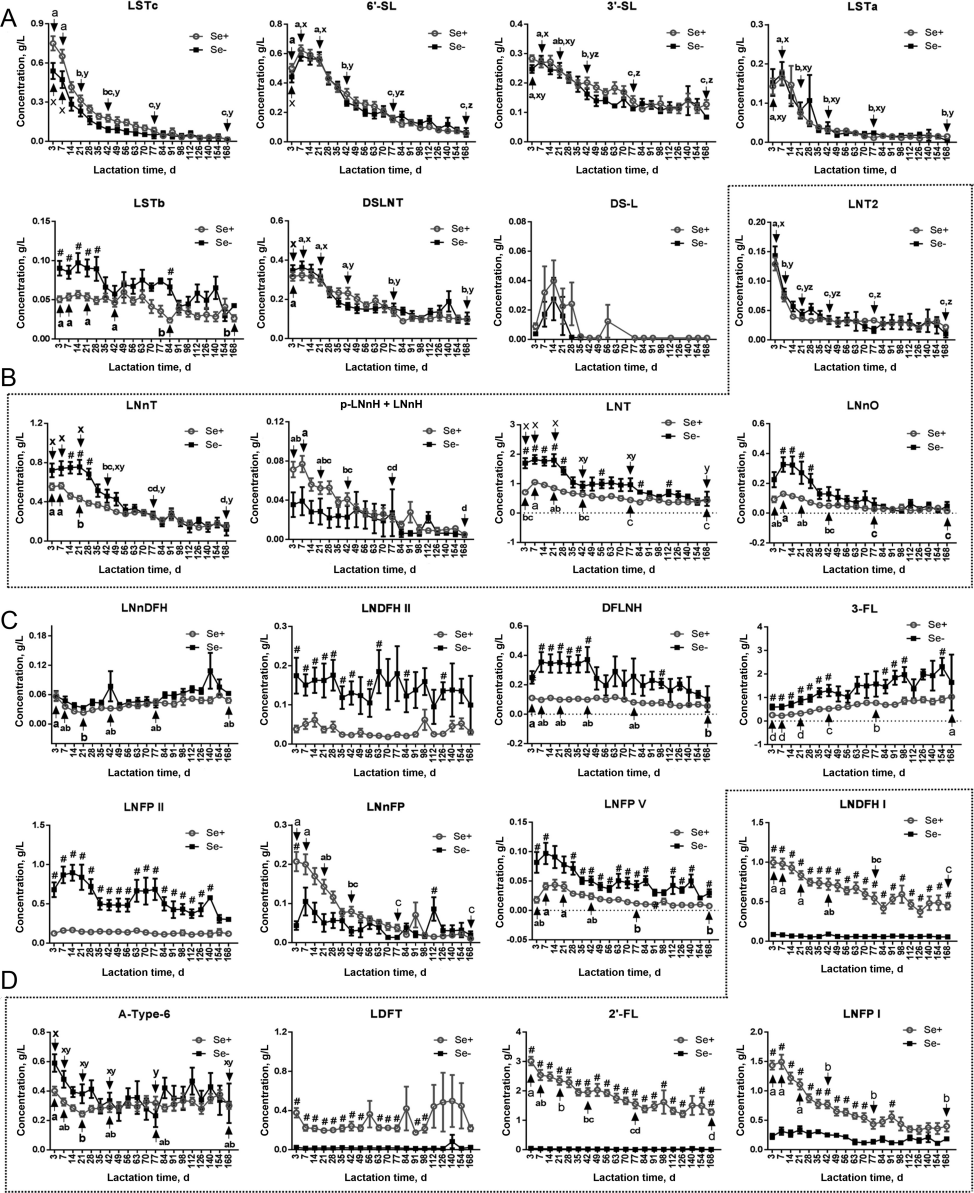
Dynamic changes of 24 HMOs in milk of Se+ and Se− mothers during lactation. Comparison of dynamic changes of (A) acidic, (B) nonfucosylated, (C) α-1-3/4-fucosylated, and (D) α-1-2-fucosylated HMOs in Se+ and Se− mothers during lactation. Acidic HMOs include LSTc, 6′-SL, 3′-SL, LSTa, LSTb, DSLNT, and DS-L. Nonfucosylated HMOs include LNT2, LNnT, p-LNnH + LNnH, LNT, and LNnO. α-1-3/4-fucosylated HMOs include LNnDFH, LNDFH II, DFLNH, 3-FL, LNFP II, LNnFP, and LNFP V. α-1-2-fucosylated HMOs include LNDFH I, A-Type-6, LDFT, 2′-FL, and LNFP I. All values are means ± SEMs. “a,” “b,” … and “x,” “y,” … indicate differences among different time points in Se+ and Se− mothers, respectively. # indicates statistical significance between Se+ and Se− mothers at the same lactation time. *P* < 0.05 was considered significant. A-Type-6, Blood Group A tetrasaccharide; DFLNH, difucosyllacto-N-hexaose; DS-L, disialyl lactose; DSLNT, disialyllacto-N-tetraose; HMO, human milk oligosaccharide; LDFT, lactodifucotetraose; LNDFH, lacto-N-difucohexaose; LNFP, lacto-N-fucopentaose; LNnDFH, lacto-N-neodifucohexaose; LNnFP, lacto-N-neofucopentaose; LNnH, lacto-N-neohexaose; LNnO, lacto-N-neooctaose; LNnT, lacto-N-neotetraose; LNT, lacto-N-tetraose; LNT2, lacto-N-triose; LST, LS-tetrasaccharide; p-LNnH, p-lacto-N-neohexaose; Se+, secretor; Se−, nonsecretor; 2′-FL, 2′-fucosyllactose; 3-FL, 3-fucosyllactose; 3′-SL, 3′-sialyllactose; 6′-SL, 6′-sialyllactose.

**Figure 3** shows the dynamic changes of all 24 HMOs in secretors and nonsecretors during lactation; concentrations on days 3, 7, 21, 42, 77, and 168 of lactation were compared to identify statistical differences during lactation (**Supplemental Figure 3**). Similarly to total acidic HMOs (Figure 2B), significant decreases were found for LS-tetrasaccharide (LST)c, 6′-sialyllactose, 3′-sialyllactose, LSTa, and disialyllacto-N-tetraose from day 3 to day 168, and no significant differences between secretors and nonsecretors were observed for these 5 individual acidic HMOs during lactation (Figure 3A). Only LSTb concentrations were significantly higher in nonsecretors than in secretors until day 28 of lactation (Figure 3A). However, compared with the other acidic HMOs, LSTb concentrations were relatively low in both secretors (<0.07 g/L) and nonsecretors (<0.1 g/L) (Figure 3A). It is worth noting that concentrations of disialyl lactose were the lowest (<0.04 g/L) and only detectable in a limited number of samples within the first month of lactation (Figure 3A).

Among nonfucosylated HMOs, LNT was most abundant in both secretors and nonsecretors (Figure 3B). In nonsecretors, mean LNT significantly decreased from 1.69 ± 0.17 g/L (day 3) to 0.48 ± 0.24 g/L (day 168); in secretors, it initially increased significantly from 0.70 ± 0.08 g/L (day 3) to 1.05 ± 0.06 g/L (day 7) but thereafter significantly decreased to 0.43 ± 0.08 g/L at day 168 (Figure 3B). From day 3 to day 28 of lactation, LNT concentrations were significantly higher in nonsecretors than in secretors, which is similar to the results for total nonfucosylated HMOs (Figures 2C, 3B). Among the other nonfucosylated HMOs, lacto-N-neotetraose (LNnT) and lacto-N-neooctaose (LNnO) were both significantly higher in nonsecretors than in secretors during early lactation (Figure 3B). In secretors, significant decreases during lactation were found for lacto-N-triose (LNT2), LNnT, p-lacto-N-neohexaose + lacto-N-neohexaose, and LNnO, whereas in nonsecretors, only LNT2 and LNnT were observed to decrease significantly (Figure 3B).

For the α-1-3/4-fucosylated HMOs, 3-FL, LNFP II, LNDFH II, difucosyllacto-N-hexaose (DFLNH), and LNFP V were all found to be significantly higher in nonsecretors than in secretors during lactation (Figure 3C). Of all 24 tested HMOs, only the mean concentration of 3-FL was observed to increase during lactation. It rose from 0.60 ± 0.11 g/L (day 3) to 2.32 ± 0.37 g/L (day 154) in nonsecretors, and from 0.25 ± 0.03 g/L (day 3) to 1.03 ± 0.08 g/L (day 168) in secretors, but statistical significance was only found in milk of secretors (Figure 3C). Other α-1-3/4-fucosylated HMOs such as LNFP II fluctuated in the range of 0.3–0.9 g/L during lactation in nonsecretors, whereas in secretors, concentrations of LNFP II were fairly low (<0.2 g/L) throughout lactation (Figure 3C). Similar results were observed for LNDFH II, DFLNH, and LNFP V in secretor and nonsecretor types during lactation (Figure 3C). Among α-1-3/4-fucosylated HMOs, only lacto-N-neofucopentaose was detected to be higher in secretors than in nonsecretors during early lactation (Figure 3C).

Among α-1-2-fucosylated HMOs, 2′-FL and LNDFH I were significantly higher in secretors than in nonsecretors throughout 168 d of lactation, whereas LNFP I and LDFT were continually significantly higher in secretors until day 91 of lactation (Figure 3D). Owing to lack of α-1-2-fucosyltransferase, all tested α-1-2-fucosylated HMOs except Blood Group A tetrasaccharide (A-Type-6) were constantly low in milk of nonsecretors (Figure 3D). In contrast, 3 α-1-2-fucosylated HMOs in secretors started highest at the beginning of lactation, with 2′-FL at 3.02 ± 0.14 g/L (day 3), LNFP I at 1.49 ± 0.12 g/L (day 7), and LNDFH I at 1.00 ± 0.06 g/L (day 3), respectively (Figure 3D). These 3 HMOs decreased significantly to 1.28 ± 0.10 g/L for 2′-FL, 0.40 ± 0.09 g/L for LNFP I, and 0.45 ± 0.04 g/L for LNDFH I at day 168 of lactation (Figure 3D). Concentrations of LDFT remained relatively stable from day 3 to day 168 of lactation in secretor mothers (Figure 3D). Concentrations and dynamic changes of A-Type-6 were similar between secretors and nonsecretors during lactation (Figure 3D).

## Discussion

In our study, the prevalence of secretors was 77% among Chinese mothers, close to the 79% secretor prevalence found by another group, which analyzed 446 milk samples from urban Chinese mothers (33). The similar results reported by these 2 independent studies indicate that the data are representative of the general population in China.

HMO profiles vary greatly by geography with different percentages of secretor and nonsecretor mothers reported worldwide (20). In Peru and California of the United States, percentages of secretors were >95% among Hispanic mothers (20). In Mexico, the secretor percentage was reportedly 100% (34). In African countries like Ghana, Ethiopia, and rural Gambia, <70% were found to be secretors (20). Compared with these populations, the percentage of secretor mothers in the Chinese population is lower than those of American populations, but higher than those seen in African populations. In particular, concentrations of 2′-FL in secretor mothers also differed considerably among geographic regions. **Table 2** summarizes 2′-FL concentrations at different lactation times reported in Asian, Western, and African populations compared with the results of Chinese mothers in the current study (22, 29, 35–39). 2′-FL concentrations were highest at the beginning of lactation despite regional differences. In Asian populations, mean concentrations of 2′-FL in colostrum were 3.02 g/L in China (current study) and 2.25 g/L in Malaysia (35) (Table 2). In Europe, mean 2′-FL concentrations in colostrum were 3.93 g/L and 4.13 g/L in Italian and German mothers, respectively (29, 37) (Table 2). In Africa, it was 1.23 g/L in colostrum from preterm South African mothers (39), and concentrations of 2′-FL in preterm and term colostrum were reportedly similar (27). This indicates that in Asian populations including Chinese mothers, 2′-FL concentrations in colostrum were lower than those of European populations but higher than those in African populations. Moreover, at days 11–30 of lactation in Chinese and Singaporean mothers, 2′-FL concentrations were 2.35 g/L and 2.17 g/L, respectively (36) (Table 2). In Europe, 2′-FL concentrations at the same lactation time were 3.02 g/L and 2.78 g/L in German and Italian mothers, respectively, whereas in America, they were 2.87 g/L and 3.5 g/L in US and Mexican mothers, respectively (22, 29, 37, 38) (Table 2). This further confirmed that 2′-FL concentrations in Asian populations were lower than those of Western populations.

**TABLE 2:** Comparison of mean 2′-FL concentrations in milk of secretor mothers of different regions around the world^1^

	Mean 2′-FL concentration, g/L					
Days of lactation	China (this study)	Malaysia (35)/Singapore (36)	Germany (37)	Italy (29)	USA (22)/Mexico (38)	South Africa* (39)
*n*	39	26/34	21	18	13/15	20
0–4	3.02	2.25/–	4.13	3.93	—	1.23
5–10	2.54	—	3.37	3.02	—	—
11–30	2.35	–/2.17	3.02	2.78	2.87/3.50	—
31–60	1.96	1.29/1.76	2.82	1.84	—	—
61–100	1.56	—	2.59	2.46	—	—

1 2′-FL, 2′-fucosyllactose.

* Data of 2′-FL concentrations in South Africa were from mothers with premature delivery.

In addition, HMO profiles are strongly influenced by secretor and nonsecretor status. The current detailed analysis of HMO profiles in Chinese mothers showed significantly higher concentrations of total HMOs in secretors than in nonsecretors during the first 2 wk of lactation, mainly due to significantly higher concentrations of neutral HMOs in secretors. Similarly, significant differences in neutral HMO concentrations were reported during early lactation but not at day 35 postpartum in Western populations (37, 40). No significant differences were observed for acidic HMOs between secretors and nonsecretors. Among neutral HMOs, in both Chinese and Western populations, α-1-2-fucosylated HMOs were significantly higher in secretors whereas α-1-3/4-fucosylated HMOs and the nonfucosylated HMOs were higher in nonsecretors (37). These differences are more obviously significant during early lactation.

HMO profiles are also influenced by lactation stages. The total concentration of HMOs dropped by one-half in both secretor (53.7%) and nonsecretor (50.1%) Chinese mothers from day 3 to day 168 of lactation. Similar trends of decreasing total HMOs were observed in Western mothers, although total HMO concentrations were reported to be higher in Western populations (37). Among neutral HMOs, decreasing concentrations of α-1-2-fucosylated HMOs and increasing concentrations of α-1-3/4-fucosylated HMOs were found in both Chinese and Western populations during lactation (37). For instance, the α-1-2-fucosylated HMO 2′-FL decreased whereas the α-1-3/4-fucosylated HMO 3-FL increased during lactation in both populations (33, 37). Interestingly, overlapping functions have been reported for these 2 HMOs. Studies have shown that 2′-FL and 3-FL share similar protective functions such as promoting probiotic growth and inhibiting pathogen adhesion (3, 41). However, properties specific to each HMO have also been observed. For instance, 1 study showed that 2′-FL, but not 3-FL, inhibited infection by *Campylobacter jejuni* (42). Therefore, studies of HMO functions and their dynamic relations will provide a more comprehensive understanding of human breast-milk glycans and help to inform more effective design of infant milk formula.

In summary, studies on the changes in HMO profiles during lactation in Chinese populations remain scarce. The current longitudinal study attempted to fill that void through intensive sampling from the same cohort of secretor and nonsecretor Chinese mothers during lactation. Twenty-four HMOs were systematically analyzed and their dynamic changes over the lactation period were reported in detail. By comparing these results with HMO results worldwide, geography and race were found to greatly influence HMO profiles. Importantly, more comprehensive studies with larger sample sizes and broader inclusion of Chinese mothers from other regions of China are still needed to understand other factors influencing HMO profiles such as diets and lifestyles. The current research project is part of an ongoing research initiative aiming to build an HMO database of Chinese mothers and to obtain a more concrete, fine-grained comparison of results from around the world. Hopefully, better knowledge on the nutritional and protective properties of HMOs in human milk will provide a strong foundation of scientific principles for guiding personalized products and precision nutrition.

## Supplementary Material

nzaa113_Supplemental_File

## Notes

Supported mainly by Quantum Hi-Tech (China) Biological Co., Ltd (to YW) and in part by Innovation Talent Program of Jiangmen City, Guangdong Province, China grant 2019ITP01 (to ZH).

Author disclosures: the authors report no conflicts of interest. All authors are employees or advisors of Quantum Hi-Tech (China) Biological Co., Ltd.

Supplemental Table 1 and Supplemental Figures 1–3 are available from the “Supplementary data” link in the online posting of the article and from the same link in the online table of contents at https://academic.oup.com/cdn/.

Abbreviations used: A-Type-6, Blood Group A tetrasaccharide; DFLNH, difucosyllacto-N-hexaose; FucT, fucosyltransferase; *FUT*, fucosyltransferase gene; HMO, human milk oligosaccharide; HPAEC, high-performance anion-exchange chromatography; LDFT, lactodifucotetraose; LNDFH, lacto-N-difucohexaose; LNFP, lacto-N-fucopentaose; LNnO, lacto-N-neooctaose; LNnT, lacto-N-neotetraose; LNT, lacto-N-tetraose; LNT2, lacto-N-triose; LST, LS-tetrasaccharide; PAD, pulsed amperometric detection; Se+, secretors; Se−, nonsecretors; 2′-FL, 2′-fucosyllactose; 3-FL, 3-fucosyllactose.
