# Supplementary material for: Systematic Characterization and Longitudinal Study Reveal Distinguishing Features of Human Milk Oligosaccharides in China
Source: Curr Dev Nutr. 2020 Jul 2;4(8):nzaa113. doi: 10.1093/cdn/nzaa113 (PMC7382630; doi:10.1093/cdn/nzaa113)
Supplement: nzaa113_Supplemental_File [file nzaa113_supplemental_file.pdf]

Systematic characterization and longitudinal study reveal distinguishing features of human milk oligosaccharides in China (Jiayi Wu)
Online Supplementary Material

Supplemental Table 1 Characteristics of studied Chinese mothers and infants.

	Charateristics	Mean ± SD[1]
Mothers	Age (years)	30.6 ± 4.3
	Weight (kg)	55.7 ± 7.4
	Height (cm)	159 ± 5
	BMI[2] (kg/m^2)	22.1 ± 2.9
	Parity	1.4 ± 0.5
	Cesarean delivery (%)	52.9
Infants	Males (%)	63.3
	Gestation age at birth (weeks)	39.0 ± 1.2

[1]Standard deviation, SD.

[2]Body Mass Index (BMI) = Weight(kg) / $Height^2(m^2)$.

Supplemental Figure 1 HMOs composition in different Lewis blood types.

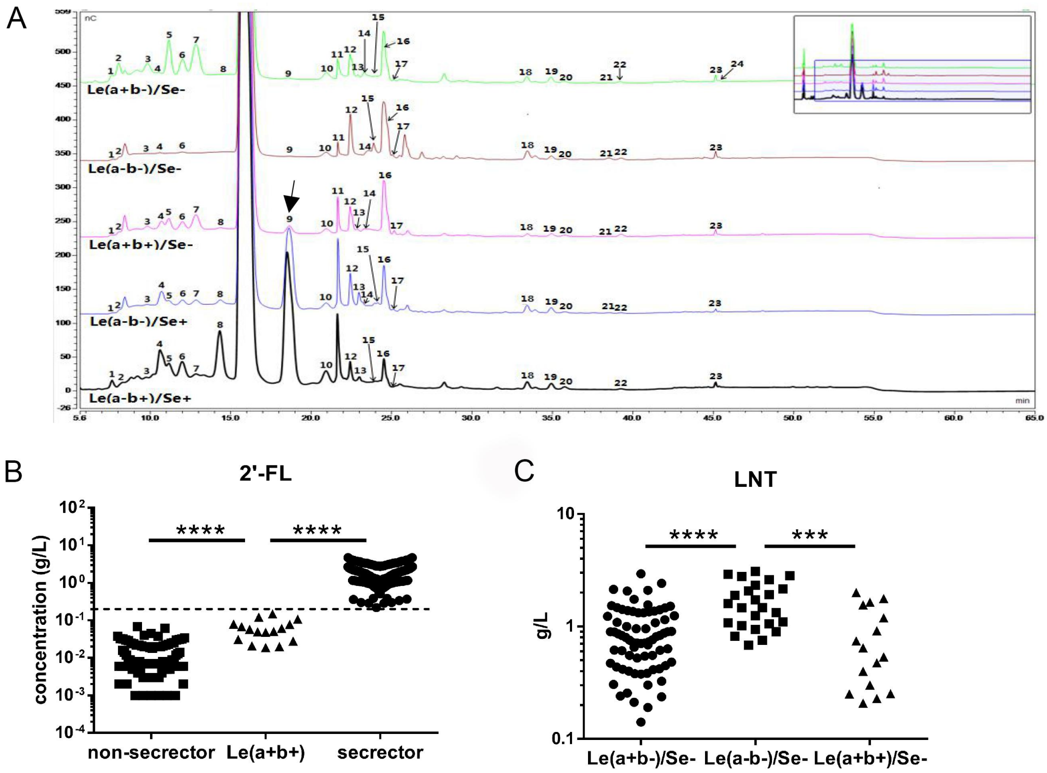

(A) Representative HPAEC chromatograms of 24 HMOs in milk samples of different Lewis blood types (Le(a±b±)) and secretor types (*Se*±). 1. LNnDFH, 2. LNDFH II, 3. DFLNH, 4. LNDFH I, 5. 3-FL, 6. A-Type-6, 7. LNFP II, 8. LDFT, 9. 2’-FL, 10. LNT2, 11. LNFP I, 12. LNnT, 13. LNnFP, 14. LNFP V, 15. p-LNnH&LNnH, 16. LNT, 17. LNnO, 18. LSTc, 19. 6’-SL, 20. 3’-SL, 21. LSTa, 22. LSTb, 23. DSLNT, 24. DS-L. (B) 2’-FL concentrations and (C) LNT concentrations in milk of different blood types (le(a±b±)) and secretor types (*Se*±). *** *P*< 0.001, **** *P*< 0.0001.

Supplemental Figure 2 Concentrations of α-1-2-fucosyltransferase products

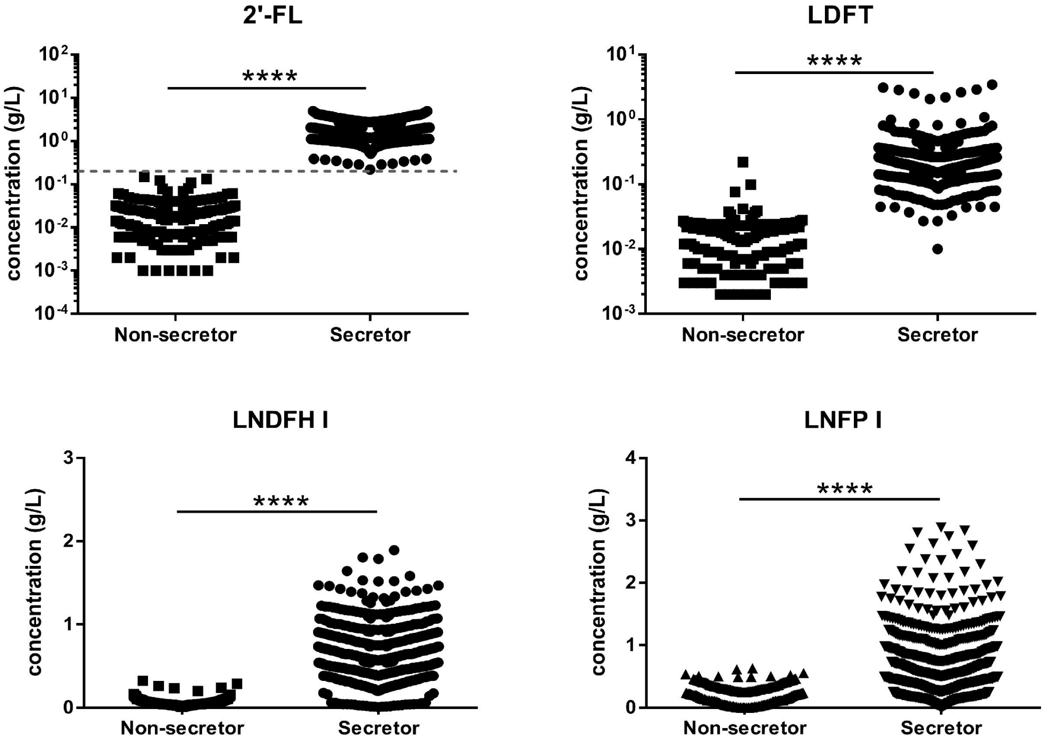

2'-FL, LDFT, LNDFH I and LNFP I in milk of defined secretors and non-secretors according to 2'-FL concentration. Dash line indicated 0.2 g/L. **** $P < 0.0001$.

Supplemental Figure 3 Concentrations (g/L) of 24 HMOs in milk of secretors and non-secretors during lactation.

A

	Day of lactation（Secretor）						Day of lactation（Non-secretor）					
	3	7	21	42	77	168	3	7	21	42	77	168
	n=25	n=27	n=25	n=15	n=10	n=7	n=13	n=12	n=10	n=10	n=4	n=2
LNnDFH	0.06±0.01a	0.04±0.00ab	0.02±0.00b	0.03±0.00ab	0.05±0.01ab	0.05±0.01ab	0.06±0.01	0.05±0.01	*0.03±0.00	0.08±0.03	0.04±0.01	0.06±0.00
LNDFH II	0.04±0.01	0.05±0.01	0.04±0.01	0.02±0.01	0.02±0.01	0.03±0.01	*0.17±0.05	**0.15±0.03	*0.16±0.04	**0.13±0.03	0.18±0.07	0.10±0.07
DFLNH	0.11±0.01a	0.10±0.01ab	0.10±0.01ab	0.10±0.01ab	0.08±0.01ab	0.06±0.00b	**0.25±0.04	**0.36±0.07	**0.35±0.07	*0.37±0.09	0.26±0.12	0.10±0.09
**3-FL**	**0.25±0.03d**	**0.23±0.02d**	**0.33±0.03d**	**0.53±0.07c**	**0.77±0.09b**	**1.03±0.08a**	****0.60±0.11**	****0.60±0.10**	****0.87±0.16**	***1.30±0.24**	**1.58±0.54**	**1.64±1.19**
**LNFP II**	**0.12±0.02**	**0.16±0.01**	**0.15±0.01**	**0.14±0.02**	**0.12±0.02**	**0.12±0.02**	*****0.68±0.09**	*****0.87±0.11**	****0.84±0.16**	****0.48±0.07**	**0.68±0.14**	***0.30±0.00**
LNnFP	0.21±0.02a	0.20±0.03a	0.14±0.02ab	0.08±0.01bc	0.04±0.01c	0.01±0.00c	***0.04±0.01	*0.11±0.04	**0.05±0.02	*0.03±0.01	*0.01±0.00	0.02±0.01
LNFP V	0.02±0.00ab	0.04±0.01a	0.04±0.01a	0.02±0.00ab	0.01±0.00b	0.01±0.00b	**0.08±0.02	*0.10±0.02	*0.08±0.01	**0.05±0.01	***0.04±0.01	**0.03±0.01
Total	0.77±0.05c	0.77±0.03c	0.79±0.04c	0.92±0.09bc	1.09±0.10b	1.30±0.09a	**1.70±0.29	**1.97±0.35	**2.22±0.43	**2.30±0.45	2.63±0.90	2.11±1.48

B

	Day of lactation（Secretor）						Day of lactation（Non-secretor）					
	3	7	21	42	77	168	3	7	21	42	77	168
	n=25	n=27	n=25	n=15	n=10	n=7	n=13	n=12	n=10	n=10	n=4	n=2
**LNDFH I**	**1.00±0.06a**	**0.98±0.07a**	**0.83±0.05a**	**0.72±0.07ab**	**0.54±0.07bc**	**0.45±0.04c**	*****0.09±0.02**	*****0.08±0.02**	*****0.07±0.01**	*****0.09±0.03**	*****0.06±0.01**	****0.06±0.01**
A-Type-6	0.40±0.03a	0.33±0.03ab	0.24±0.02b	0.29±0.03ab	0.32±0.04ab	0.30±0.02ab	**0.59±0.06x	**0.48±0.06xy	0.38±0.06xy	0.33±0.05xy	0.23±0.08y	0.32±0.13xy
LDFT	0.38±0.05	0.22±0.03	0.20±0.02	0.24±0.05	0.22±0.03	0.22±0.03	***0.02±0.01	***0.01±0.00	***0.02±0.00	***0.02±0.00	**0.02±0.01	*0.02±0.00
**2'-FL**	**3.02±0.14a**	**2.54±0.18ab**	**2.35±0.15b**	**1.96±0.14bc**	**1.56±0.15cd**	**1.28±0.10d**	*****0.05±0.01**	*****0.04±0.01**	*****0.02±0.01**	*****0.02±0.01**	*****0.01±0.00**	*****0.01±0.01**
**LNFP I**	**1.44±0.08a**	**1.49±0.12a**	**1.11±0.09a**	**0.77±0.07b**	**0.44±0.08b**	**0.40±0.09b**	*****0.23±0.04**	*****0.31±0.06**	*****0.34±0.06**	*****0.25±0.04**	***0.13±0.04**	**0.18±0.03**
Total	6.23±0.19a	5.56±0.34ab	4.73±0.23bc	3.98±0.23cd	3.08±0.25de	2.64±0.18e	***0.97±0.07x	***0.92±0.09x	***0.82±0.07xy	***0.68±0.09xy	***0.45±0.09y	**0.58±0.15xy

C

	Day of lactation（Secretor）						Day of lactation（Non-secretor）					
	3	7	21	42	77	168	3	7	21	42	77	168
	n=25	n=27	n=25	n=15	n=10	n=7	n=13	n=12	n=10	n=10	n=4	n=2
LNT2	0.13±0.01a	0.07±0.01b	0.04±0.00c	0.03±0.00c	0.03±0.00c	0.02±0.00c	0.14±0.02x	0.08±0.01y	0.04±0.01yz	0.03±0.01yz	*0.02±0.01z	*0.01±0.01z
LNnT	0.55±0.03a	0.56±0.03a	0.43±0.03b	0.34±0.03bc	0.25±0.03cd	0.15±0.02d	*0.72±0.07x	*0.74±0.07x	**0.76±0.07x	0.46±0.07xy	0.27±0.03y	0.12±0.06y
LNnH#	0.07±0.01ab	0.08±0.01a	0.05±0.01abc	0.04±0.01bc	0.02±0.00cd	0.01±0.00d	*0.04±0.01	*0.04±0.01	0.03±0.01	0.02±0.01	0.03±0.02	0.00±0.00
**LNT**	**0.70±0.08bc**	**1.05±0.06a**	**0.85±0.06ab**	**0.63±0.08bc**	**0.44±0.08c**	**0.43±0.08c**	*****1.69±0.17x**	*****1.82±0.15x**	****1.80±0.21x**	**0.91±0.15xy**	***0.96±0.22xy**	**0.48±0.24y**
LNnO	0.09±0.02ab	0.13±0.02a	0.10±0.01ab	0.05±0.01bc	0.03±0.01c	0.02±0.01c	*0.23±0.05	**0.33±0.05	*0.27±0.07	0.13±0.05	0.06±0.04	0.05±0.03
Total	1.54±0.11b	1.89±0.08a	1.47±0.08b	1.10±0.10c	0.78±0.11de	0.62±0.09e	***2.82±0.22x	***3.00±0.20x	***2.89±0.23x	1.55±0.22y	*1.34±0.28y	0.66±0.20y

D

	Day of lactation（Secretor）						Day of lactation（Non-secretor）					
	3	7	21	42	77	168	3	7	21	42	77	168
	n=25	n=27	n=25	n=15	n=10	n=7	n=13	n=12	n=10	n=10	n=4	n=2
LSTc	0.75±0.05a	0.65±0.05a	0.32±0.04b	0.18±0.03bc	0.08±0.02c	0.01±0.00c	*0.54±0.06x	*0.47±0.06x	0.23±0.04y	*0.09±0.02y	0.05±0.03y	0.01±0.00y
6'-SL	0.50±0.03a	0.63±0.03a	0.56±0.04a	0.32±0.04b	0.15±0.02c	0.06±0.01c	0.44±0.04x	0.59±0.04x	0.56±0.05x	0.27±0.02y	0.16±0.02yz	0.06±0.03z
3'-SL	0.28±0.01a	0.27±0.02a	0.24±0.01ab	0.20±0.02b	0.14±0.02c	0.13±0.02c	0.25±0.01xy	0.27±0.02x	0.24±0.03xy	0.16±0.02yz	0.11±0.00z	0.08±0.00z
LSTa	0.14±0.02a	0.17±0.02a	0.08±0.01b	0.03±0.01b	0.01±0.00b	0.02±0.00b	0.15±0.03xy	0.18±0.03x	0.08±0.02xy	0.04±0.01xy	*0.02±0.00xy	0.00±0.00y
LSTb	0.05±0.00a	0.05±0.00a	0.05±0.00a	0.05±0.01a	0.04±0.01ab	0.03±0.00b	***0.09±0.01	**0.09±0.01	**0.09±0.01	0.06±0.01	**0.07±0.00	0.04±0.00
DSLNT	0.32±0.02a	0.32±0.03a	0.30±0.03a	0.23±0.03a	0.14±0.02b	0.10±0.01b	0.35±0.03x	0.36±0.03x	0.31±0.04x	0.16±0.02y	0.15±0.03y	0.10±0.03y
DS-L	0.01±0.00	0.03±0.02	0.02±0.01	0.00±0.00	0.00±0.00	0.00±0.00	0.00±0.00	0.02±0.01	0.02±0.01	0.00±0.00	0.00±0.00	0.00±0.00
Total	2.05±0.10a	2.09±0.10a	1.55±0.09b	1.01±0.09c	0.56±0.07d	0.33±0.03d	1.82±0.10x	1.97±0.11x	1.53±0.11x	*0.77±0.06y	0.57±0.02y	0.30±0.02y

Changes of 24 HMOs as groups in subtypes (A) acidic HMOs, (B) non-fucosylated HMOs, (C) α-1-3/4-fucosylated HMOs and (D) α-1-2-fucosylated HMOs on day3, 7, 21, 42, 77 and 168 during lactation. All values are means ± SEMs. “LNnH#” was a mixture of p-LNnH & LNnH. “a”, “b”, ... and “x”, “y”, ... indicated differences among different time points in secretors and non-secretors, respectively. Asterisks indicated statistical significance between secretors and non-secretors at the same lactation time. * $P<0.05$, ** $P<0.01$, and *** $P<0.001$.
